# Comet assay based DNA evaluation of fuel filling stations and automobile workshops workers from Khyber Pakhtunkhwa province, Pakistan

**DOI:** 10.1186/s12995-015-0069-2

**Published:** 2015-08-01

**Authors:** Muhammad Khisroon, Aisha Gul, Ajmal Khan, Naheed Ali, Farah Zaidi, Syed Basit Rasheed, Ahmadullah, Huma Akbar

**Affiliations:** Department of Zoology, University of Peshawar, Peshawar, Pakistan; Institute of Chemical Sciences, University of Peshawar, Peshawar, Pakistan

**Keywords:** Gasoline hydrocarbons, DNA damage, Comet assay, Blood lymphocytes

## Abstract

**Background:**

Fuel filling stations workers and automobile workshops mechanics are consistently exposed to gasoline hydrocarbons during their occupation, this may cause DNA damage. Objective of this study was to evaluate the level of DNA damage in subjects occupationally exposed to these hydrocarbons.

**Methods:**

Comet assay was performed on blood lymphocytes of exposed subjects to assess the probable DNA damage. 100 cells per individual were scored and graded by comet tail length. Exposed group consisted of 98 subjects (age 25.4 ± 7.2 years), of which 68 were CNG/Petrol filling men and 30 were automobile workshop workers, selected randomly from different service stations and automobile workshops of populated and adjacent cities of Peshawar, Mardan and Nowshera of Khyber Pakhtunkhwa province, while control group included 92 subjects (age 26.7 ± 11.8 years) were also from the same areas.

**Results:**

Significantly high level of DNA damage was found in the subjects exposed to gasoline hydrocarbons as compared to control subjects (173.2 ± 50.1 and 61.0 ± 25.0, *P* = 0.001, respectively). Period of exposure and use of tobacco also showed considerable effects (P < 0.05) on DNA damage, while effect of age and daily working hours on total comet score (TCS) were non-significant (*P* > 0.05).

**Conclusions:**

The results of our study concluded that petroleum hydrocarbons have the potential to cause DNA damage in the exposed subjects. The study also suggested that protective strategies should be implemented by the concerned authorities to minimize exposure to fuel hydrocarbons.

## Background

Gasoline (Petrol) is a complex man-made compound that does not exist naturally in the environment. Most of its chemicals are present around the human settlement in a number of physical states i.e. liquid, gaseous or in some other form. Gasoline is extracted in the refining process of crude petroleum [1]. Liquid gasoline in petroleum chemicals is one of the known complex mixtures to which human is exposed. It contains more than 150 hydrocarbons with a boiling range of 40 °C to 180 °C. Hydrocarbons of gasoline are alkanes (paraffins), isoparaffins, alkenes (olefins) and naphthenics in a ratio of about 50 % to 70 %, while its aromatic compounds are benzene, toluene, ethylbenzene and xylene, present in a ratio of 30 % to 40 %, all of which are significantly hazardous carcinogenic chemicals. More than 1,000 possible chemical substances are found in gasoline [2].

The significant release of vapours from gasoline resulted in direct human exposure to these vapours at various contact points and most importantly in the large number of retail service stations and adjoining settled areas [3]. The vapour form of gasoline, when present in the atmosphere can dispensed at any time, especially at fuel filling service stations, so the people who are working in fueling and refueling of vehicles are more exposed and are at higher risk to its adverse health effects [4].

A number of epidemiological and experimental studies have shown that fumes of petrol and diesel engines are carcinogenic and mutagenic to both humans and animals [5]. All places where refinery products are manufactured, processed or soled are potentially carcinogenic to humans [6].

Benzene a widely spread environmental pollutant, constitutes about 1–5 % of petrol [7]. It is classified as a known carcinogen to human [6]. Persons working at filling stations, motor mechanic, tanker crew and traffic personnel are more susceptible to benzene exposure due to their occupation [8]. Benzene also affects hematopoiesis in the bone marrow [9]. It lowers the number of peripheral white blood cells and platelet, specifically decreases T-cells, B-cells and granulocytes [10]. Due to metabolism of benzene in human liver, its metabolites repress the production of DNA, RNA and various other cellular proteins [11, 12]. Due to its clastogenic property benzene can produce disorders like micronuclei, chromosomal aberrations and sister chromatid exchange [13]. Thus for the improvement of health and occupational safety, monitoring of occupational exposure to chemicals is important both for the evaluation of risks and for the implementation of strategies [14].

A number of techniques such as sister chromatid exchange, chromosomal aberration and micronucleus assay are normally used for investigating genetic damages. However, these methods are economically costly, time consumable and require proliferating cells. Therefore, the use of single cell gel electrophoresis (SCGE) or comet assay for genotoxicity studies have greatly increased during the past few decades [15–17].

Considering the hazardous effects of gasoline hydrocarbons and lack of awareness among our study group, the aim of present study was to assess the level of DNA damage in retail service stations attendants and automobile workshops mechanics and of corresponding unexposed control subjects.

## Methods

### Study population

The study population was consisted of 190 people, of which 98 were exposed subjects and 92 control group. Among the exposed subjects, 68 were CNG/Petrol filling men while 30 were automobile workshops workers, selected from different retail service stations and automobile workshops located in Peshawar, Nowshera and Mardan cities of Khyber Pakhtunkhwa province. All exposed subjects were males with mean age of 25.4 ± 7.2 years. All the subjects were healthy, with no history of diseases, and were not taking any kind of medications which could cause the DNA damage. Control group was also selected from the same area with mean age of 26.7 ± 11.8 years and no exposure to petroleum fumes or other potentially genotoxic substances. The study was approved from Ethical Committee of the Department of Pharmacy, University of Peshawar (42/Pharm, Dated: 12.05.2014).

### Questionnaire

Information were collected from all the subjects on a questionnaire according to their own consent, which included demographic data of the subjects, medical history, addiction of tobacco or alcohol and complete details of their occupation i.e. daily duty hours, years of exposure, use of protective devices etc. Samples were collected from all the subjects at their workplaces on the working days.

### Blood sample collection and lymphocyte separation

3 ml of venous blood sample was collected from each subject using sterilized syringes and then transferred to K-EDTA containing tubes. The samples were labeled, transported to the laboratory and processed within 3 to 4 h. Lymphocytes were isolated from the blood by Ficoll-1077 density gradient centrifugation and washed in phosphate buffered saline (PBS). The viability of the cells was tested by Trypan blue and was kept greater than 90 %.

### Alkaline comet assay

We used the standard procedure of alkaline comet assay described by Singh et al. [15] with slight modifications. Duplicate slides per sample were prepared. For the preparation of pre-coated slides, the conventional glass slides were dipped into hot normal melting agarose (NMA) (0.7 %), laid in a tray to air dry and then wiped from the underside to remove the extra agarose. Slides were generally prepared one day before use, labeled and then stored at room temperature. 15 μl of cell suspension was mixed with 70 μl of low melting point agarose (LMPA) (0.7 %), spread on top of pre-coated slides and kept at 0 °C for 5 min with cover slip on it. After that the cover slip was removed and a second layer of 85 μl LMPA was added to fill any residual holes and again kept at 0 °C for 5 min to solidify, with cover slip on it.

### Lysing of slides

After solidification, the cover slips were removed and slides were gently immersed in freshly prepared cold-lysing solution [(2.5 M NaCl, 100 mM Na_2_EDTA, 10 mM Tris, pH 10) with 1 % Triton X-100 and 10 % DMSO added just before use] for at least 2 h at 4 °C.

### Electrophoresis and neutralization

After lysing, the slides were immersed in electrophoresis buffer (300 mM NaOH and 1 mM EDTA, pH 13) and left for 20 min to allow the unwinding of DNA and expression of alkali-labile sites. The slides were then subjected to electrophoresis for 25 min at 300 mA and 25 V. To prevent any kind of unintentional DNA damage, the slides were protected from direct exposure to light. The steps were conducted at 4 °C. After electrophoresis, the slides were neutralized by washing three times with neutralization buffer (400 mM Tris, pH 7.5) for 5 min each.

### Staining, scoring and visualization of slides

**Fig. 1:**
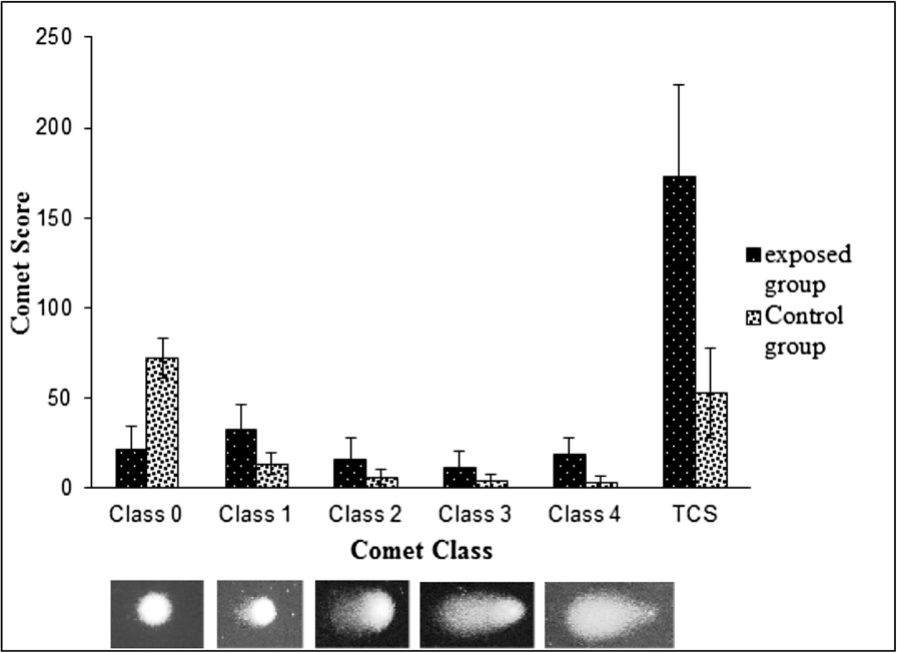
DNA damage assessed using the comet assay. Mean comet score and comet classes in control and exposed groups. The cells were assessed visually and received class 0 (undamaged) to 4 (maximally damaged), according to the size and shape of the tail. Score were obtained using the mean score of three independent, blind evaluators

The slides were stained with 70 μl Acridine orange dye (20 μg/ml) and kept for 5 min. Cover slips were placed on it, and viewed at 200x of fluorescent microscope (Nikon Eclipse 80 i) equipped with 450-490 nm excitation filter. In order to calculate DNA damage, 100 cells per sample were chosen randomly and analyzed visually according to comet appearance. 5 classes, i.e. from class 0 (no DNA damage) to class 4 (maximum DNA damage) give sufficient declaration (Fig. 1). Visual scoring is a reliable, simple and rapid method for scoring the comets [18]. Total comet score (TCS) was then calculated according to the formula, TCS = 0(n) +1(n) +2(n) +3(n) +4(n), where “n” indicates number of cells in each class [18].

### Statistical analysis

Statistical analysis was performed, using SPSS V.20.0. Mean and standard deviation values of the data were determined. Student’s *t*–test (two-tailed) was used to calculate mean differences, where P value was kept at 0.05 for statistical significance. The comet assay data, which was non-normally distributed, were analyzed using non-parametric Mann-Whitney *U*-test (two-tail). Correlation was calculated for the duration of occupational exposure and TCS using online software [19].

## Results

The effect of occupational exposure due to petroleum hydrocarbons on the DNA in fuel filling station/automobile workers and control subjects was assessed by the comet assay. The main characteristics including age, tobacco consumption, duration and nature of occupation of the studied population are given in Table 1. The age of exposed and control groups had no significant difference (i.e. *P* > 0.05). The exposed and control subjects were healthy having no such disease which could cause DNA damage.

**Table 1:** Distribution of the main characteristics of persons working in CNG/petrol pumps and automobile workshops and control group

Main characteristics	Exposed group	Control group
N	98	92
Mean Age (Years)	25.4 ± 7.2	26.7 ± 11.8
Mean Duration of Job (Years)	4.4 ± 4.8	
Nature of Job		
Fuel filling station workers	68 (69.4 %)	
Automobile Workshop workers	30 (30.6 %)	
Smoking		
No	71 (72.4 %)	70
Yes	27 (27.6 %)	22

N, Number of subjects

The mean total comet score (TCS) in 100 lymphocytes of study group is shown in Table 2. The cells with DNA damaged had the appearance of a comet, while undamaged cells had an intact nucleus without a tail (Fig. 1). DNA damage observed in persons working in CNG/ Petrol pumps, and automobile workshops (TCS = 173.2 ± 50.1) were significantly higher than that observed in the control group (TCS = 61.0 ± 25.0, *P* ≤ 0.001) (Table 2). Comet class 3 (11.3 ± 9.9 cells) and class 4 (18.7 ± 9.4 cells) were observed more frequently in exposed group than in the control group (5.3 ± 4.1 and 3.7 ± 2.3 cells, respectively). The opposite results were observed with undamaged cells; comet class 0 was observed more frequently in the control group (67.7 ± 13.0) as compared to the persons working in CNG/ Petrol Pumps, and automobile workshops (21.6 ± 13.4 cells) (Table 2). Figure 1 shows the graphical representation of the mean TCS and Comet classes in both persons working in CNG/Petrol pumps, and automobile workshops and control groups.

**Table 2:** Mean frequency of each comet class per 100 cells (± standard deviation) and overall mean comet score (± standard deviation) of persons working in CNG/petrol pumps, automobile workshops (exposed group) and control group

Comet class	0	1	2	3	4	TCS
Exposed group	21.6 ± 13.4	32.5 ± 14.2	15.8 ± 11.9	11.3 ± 9.9	18.7 ± 9.4	173.2 ± 50.1*
Control group	67.7 ± 13.0	16.2 ± 8.0	7.1 ± 4.4	5.3 ± 4.1	3.7 ± 2.3	61.0 ± 25.0

*Difference significant relative to control group at *P* ≤ 0.001 (student’s t-test); TCS, total comet score

A positive correlation (*r* = 0.96, *P* ≤ 0.02) was observed between the duration of occupational exposure and TCS. Among the study group, the lowest TCS value (160.7 ± 42.0) was observed in the subject working for less than a year, while the exposed individuals who worked for 1 to 5 years had the TCS of 173.5 ± 52.4. The highest TCS value (183.7 ± 45.9) was observed in those working for more than 6 to 10 years (Table 3). The results indicate that the TCS increased with increasing exposure duration of job.

**Table 3:** Comet score distributed according to duration of occupational exposure

Duration	N	TCS
<1 year	20	160.7 ± 42.0
1 to 5 years	52	173.5 ± 52.4
6 to 10 years	26	183.7 ± 45.9

*r* = 0.96, *P* ≤ 0.02

No significant effect of age and per day exposure hours on TCS was observed. The effect of smoking on TCS value was evaluated.

Smoking habits had significant effect on TCS value among the exposed and the control subjects (Table 4). A significant increase (*P* ≤ 0.001) in TCS values was observed in smokers when compared with non-smokers in both control and exposed groups (Table 4).

**Table 4:** Effect of tobacco use on TCS (mean ± SD)

Subject	N	TCS
*Control*		
Smokers	22	71.6 ± 23.2
Nonsmokers	70	53.0 ± 25.0
*Exposed*		
Smokers	27*	177.0 ± 51.3
Nonsmokers	71*	143.0 ± 46.5

*Difference significant relative to control group at *P* ≤ 0.001 (Mann–Whitney *U*-test, two-tail)

**Table 5:** TCS (mean ± SD) according to nature of job in CNG/petrol pumps and automobile workshops

Nature of Job	N	TCS
Automobile workshop workers	30	204.0 ± 59.3
CNG/Petrol filling men	68	169.7 ± 48.1

*P* ≤ 0.001; SD, standard deviation

The automobile workshop mechanics had significantly higher TCS (204.0 ± 59.3, P = 0.001) than CNG/Petrol pumps filler men (169.7 ± 48.1) (Table 5).

## Discussion

According to petroleum industry the highest occupational exposure to gasoline vapours typically occurs amongst marine loading operators, truck drivers, service station attendants and bulk terminal operators [20]. Retail service stations attendants are workers who experience chronic exposure to petroleum products mainly through inhalation due to their occupational exposure [21]. As these workers are exposed to gases such as CO_2_, CO, NO_2_, Hydrocarbons, Nitro aromatics, Benzopyrene, Benzene, 1, 3 Butadiene etc, all these gases are chemical mutagens [22]. Similarly in engine repair workshops engine exhaust and used engine oils are also major sources of polycyclic aromatic hydrocarbons (PAH) [23].

The Current research work showed a significant DNA damage in the exposed subjects as compared to control group. This study correlates with the study done in Thailand, which showed greater DNA damage in fifty workers of gasoline stations than that in control group [24]. Santos-Mello et al. [25] have demonstrated hematopoietic malignancy and chromosomal deletions in lymphocytes of workers exposed to petrol. In a study from North India, genotoxicity in retail service stations attendants showed significantly higher level of DNA damage in exposed subjects as compared to control subjects [26]. Another study from India showed higher level of genotoxicity in filling station attendants exposed to petroleum hydrocarbons as compared to control group [27].

We observed an increase in TCS with an increase in exposure duration. Similarly Schnatter et al. [28] found a close relationship between duration of occupation and genotoxicity. Keretetse et al. [29] in their pilot study on petrol attendants showed that subjects exposed for 5-10 years or >10 years have the highest ratio of DNA damage. Subjects exposed for 1-3 years also had increased DNA damage as compared to those exposed for less than a year. A research work done by Shastri and Pant [30] claimed that occupational exposure to the chemicals such as heavy metals, contained in brake fluids, degreasers, detergents, lubricants, metal cleaners, paints, fuel, solvents, found in the garage environment, can cause DNA damage and the genotoxic effects increases with increase in duration of exposure. On the other hand the study conducted by Rekhadevi et al. [27] showed no significant association with the duration of exposure.

Smoking is one of the first exposure parameters to which researchers give their attention, as this is an agent that has been supposed to produce harmful effects [31]. Tobacco consumption affected the results for DNA damage used in this study, a significant increase in TCS values was observed in smokers when compared with non-smokers in both control and exposed groups (Table 4). Similarly, from Italy a research on 200 individuals showed that the extent of DNA migration increased up to 10 % in lymphocytes of smokers, but they detected no effect or no relationship to the amount of cigarettes being smoked per day [32]. Many other occupational studies from France [33], Poland [34] and Turkey [35, 36] have also reported damaging effects of smoking. A study from China revealed that in a cigarette factory both employees and smokers had more DNA damage as compared to control non-smokers that were not occupationally exposed to any kind of tobacco dust [37].

Several earlier epidemiological studies have reported that gasoline stations attendants and garage mechanics have an increased risk of haematological malignancy. In Washington State, Milham [38] analyzed death certificates (from 1950-79) of four occupational groups with petrol or fuel exposures, and have found higher risks for numerous haematopoietic cancers. Car mechanics were found to have significantly higher level of lymphatic leukemia, and other lymphomas, and non-significantly higher level of Hodgkin’s disease, unspecified leukemia and acute leukemia. The workshop mechanics continuously wash their hands with petrol during car or engine repair; therefore these workers inhale petrol gases which contain genotoxic substances, like benzene and the products derived from engine ignition. Hadnagy and Seemayer [39] showed that, when these products enter the blood circulation cause genotoxic and cytotoxic properties. Karahalil et al. [40] used sister chromatid exchange test and micronucleus assay on peripheral blood lymphocytes of oil and petrol engine repair garages workers. The exposed group showed a significant high (P < 0.05) value of Micronuclei as compared to control group i.e. 1.87 ± 0.04 and 1.56 ± 0.06, respectively. These investigations are in line with our results, in which the automobile workshop mechanics showed higher level of DNA damage as compared to fuel filling station attendants.

## Conclusions

Pakistan is a developing country and there are no provisions for the safe use of petroleum at work places in Pakistan. As a result the workers’ health is negatively affected. The study results concluded that gasoline and its various chemicals impose genotoxic effects and cause increase DNA damage in exposed workers during their occupational exposure, as compared to control. This DNA damage may be attributed to the extensive exposure to petrol without any protective strategy.

## Abbreviations

CA: Comet assay
CNG: Compressed natural gas
DMSO: Dimethyl sulfoxide
EDTA: Ethylenediaminetetraacetic acid
IARC: International Agency for Research on Cancer
LMPA: Low melting point agarose
MN: Micronuclei
NMA: Normal melting agarose
PBS: Phosphate buffered saline
SCGE: Single cell gel electrophoresis
TCS: Total comet score
